# Convolution, Correlation and Generalized Shift Operations Based on the Fresnel Transform

**DOI:** 10.3390/s23031663

**Published:** 2023-02-02

**Authors:** Juan M. Vilardy, Eder Alfaro, Johonfri Mendoza

**Affiliations:** 1 Grupo de Investigación en Física del Estado Sólido (GIFES), Faculty of Basic and Applied Sciences, Universidad de La Guajira, Riohacha 440007, Colombia; 2 Grupo de Investigación en Física del Estado Sólido (GIFES), Faculty of Engineering, Universidad de La Guajira, Riohacha 440007, Colombia

**Keywords:** Fresnel transform, shift operation, convolution, correlation, optical systems

## Abstract

The Fresnel transform (FrT) is commonly used to describe the free-space propagation of optical waves. In this work, we present new definitions for the convolution, correlation and generalized shift operations based on the FrT. The generalized shift operation is defined by using simultaneous space and phase shifts. The generalized shift operation is useful for centred optical systems in the Fresnel domain (FrD) when the data distributions at the input plane of the optical system are shifted. The new convolution and correlation operations defined in terms of the FrT, the wavelength and the propagation distance, can be considered as a generalization of the usual convolution and correlation operations. The sampling theorem for distributions, whose resulting FrT has finite support, is formulated by using the new convolution operation introduced in this work and a new definition of the Dirac comb function. These new definitions and results could be applied to describe, design and implement optical processing systems related to the FrT. Finally, we present a centred optical systems used in holography and optical security systems that can be described or modelled by the new definitions of the operations proposed in this paper.

## 1. Introduction

The Fresnel approximation of the general scalar diffraction theory is a reduction of the Huygens–Fresnel principle that explains the free-space propagation of optical waves [1]. This Fresnel approximation is known as the Fresnel diffraction or Fresnel transform (FrT). The FrT and the operations of shift, modulation, convolution and correlation have been commonly used in several optical information processing systems, such as holographic systems, optical wavefront modulation, image processing and optical communications systems, among others [1,2,3,4,5,6,7,8]. The operations of shift, convolution and correlation have been proposed in the Gyrator domain [9] and the fractional Fourier domain [10,11] with the purpose of developing several information processing applications based on the Gyrator transform and the fractional Fourier transform, respectively. These applications are the sampling theorem, filtering, interpolation, encryption, decryption, authentication and pattern recognition, among others.

In this work, we introduce new definitions for the generalized shift, convolution and correlation operations based on the FrT. The generalized shift operation is a shift and a modulation by a pure linear phase of a given function, and the FrT of this generalized shift allows us to obtain a function in the Fresnel domain (FrD) without shift. The proposed definitions for the convolution and correlation operations at the spatial domain are explicit integral expressions that depend on the parameter λ and *z*, which are the wavelength of illumination and the distance propagation of the FrT, respectively. These integral expressions at the spatial domain can be considered as generalizations of the usual convolution and correlation operations. The new convolution and correlation operations in the FrD are performed by the product of two FrTs and a quadratic phase term. We develop and present two applications based on the new operations in the FrD. The first application is the sampling theorem for functions whose resulting FrTs have finite support, and the second application is the proposal of a modified version of the double random phase encoding (DRPE) in the FrD with an improvement to security over the encrypted distribution [12].

The paper is organized as follows: In Section 2, the definition and properties of the FrT are presented. In Section 3, the new operations based on the FrT are defined. Two applications of the new operations based on the FrT are developed in Section 4. The results presented and discussed in this work are summarized in Section 5 in the conclusions.

## 2. Fresnel Transform (FrT) and Properties

The Fresnel transform (FrT) corresponds to the free-space propagation of an object f(x) when it is illuminated by a plane wave of wavelength λ for a propagation distance *z*. Therefore, the FrT is defined by [1]

(1) $$\begin{array}{c} f_{z}\left(u\right)={\mathrm{FrT}}_{\lambda ,z}\left\{f\left(x\right)\right\}=\int_{-\infty }^{+\infty }f\left(x\right)h_{\lambda ,z}\left(u,x\right)dx, \end{array}$$

and the kernel $h_{\lambda ,z}\left(u,x\right)$ of the FrT is

(2) $$h_{\lambda ,z}\left(u,x\right)=M_{\lambda ,z}\exp\left\{\frac{i\pi }{\lambda z}{\left(u-x\right)}^{2}\right\},\;\mathrm{and}\;M_{\lambda ,z}=\frac{1}{\sqrt{i\lambda z}}\exp\left\{i\frac{2\pi z}{\lambda }\right\},$$

where *x* and *u* denote the coordinates at the spatial domain and the Fresnel domain (FrD), respectively, and $M_{\lambda ,z}$ is a constant term for a given wavelength λ and a distance of propagation *z*. The expression ${\mathrm{FrT}}_{\lambda ,z}$ represents the FrT for parameters λ and *z*. The inverse FrT corresponds to the FrT for parameters λ and −z. The main properties of the FrT, which are fundamental to the following sections, are

(3) $${\mathrm{FrT}}_{\lambda ,z_{1}}\left\{{\mathrm{FrT}}_{\lambda ,z_{2}}\left[f\left(x\right)\right]\right\}={\mathrm{FrT}}_{\lambda ,z_{1}+z_{2}}\left\{f\left(x\right)\right\},$$

(4) $${\mathrm{FrT}}_{\lambda ,z}\left\{f(x-x_{0})\right\}=f_{z}\left(u-x_{0}\right),$$

(5) $${\mathrm{FrT}}_{\lambda ,z}\left\{\exp\left(\frac{i2\pi v_{0}x}{\lambda z}\right)f\left(x\right)\right\}=\exp\left\{\frac{i\pi }{\lambda z}\left(2v_{0}u-v_{0}^{2}\right)\right\}f_{z}\left(u-v_{0}\right),$$

where $x_{0}$ and $v_{0}$ are real constants. Equation (3) shows that the FrT is additive with respect to the parameter *z*. When a shift $x_{0}$ is introduced at the spatial domain for the object f(x), the result of its FrT is also shifted in the FrD by the same amount of shift $x_{0}$ given by Equation (4). Finally, Equation (5) shows that the FrT of an object f(x) modulated by a pure linear phase term produces a shift and a modulation by another pure linear phase at the FrD. If we combine Equations (4) and (5) and define $v_{0}=-x_{0}$, the following important property of the FrT is obtained [3,4,5]:

(6) $${\mathrm{FrT}}_{\lambda ,z}\left\{\exp\left(\frac{-i2\pi x_{0}x}{\lambda z}\right)f\left(x-x_{0}\right)\right\}=\exp\left\{\frac{-i\pi }{\lambda z}\left(2x_{0}u+x_{0}^{2}\right)\right\}f_{z}\left(u\right).$$

## 3. New Processing Operations Based on the FrT

### 3.1. Generalized Shift Operation

We use the result of Equation (6) in order to propose a new generalized shift operation given by a shift and a modulation by a pure linear phase for an object f(x). This new generalized shift operation with parameters $x_{0}$, λ and *z* is proposed as:

(7) $${\mathrm{GS}}_{x_{0};\lambda ,z}f\left(x\right)=\exp\left\{\frac{-i2\pi x_{0}}{\lambda z}\left(x-\frac{x_{0}}{2}\right)\right\}f\left(x-x_{0}\right).$$

The generalized shift operation is additive with respect to the parameter $x_{0}$, as follows ${\mathrm{GS}}_{x_{1};\lambda ,z}{\mathrm{GS}}_{x_{2};\lambda ,z}={\mathrm{GS}}_{x_{1}+x_{2};\lambda ,z}$. The generalized shift operation becomes the usual shift operation ${\mathrm{GS}}_{x_{0};\lambda ,z}f\left(x\right)=f\left(x-x_{0}\right)$ when the parameter *z* is too large or tends to infinity. The FrT for parameters λ and *z* of the new generalized shift operation is computed by using the result of Equation (6):

(8) $${\mathrm{FrT}}_{\lambda ,z}\left\{{\mathrm{GS}}_{x_{0};\lambda ,z}f\left(x\right)\right\}=\exp\left\{\frac{-i2\pi x_{0}u}{\lambda z}\right\}f_{z}\left(u\right).$$

The resulting FrT for the new generalized shift operation does not introduce a shift in the FrD. The right combination of Equations (4) and (5), which is represented by the proposed definition of our new generalized shift operation, can be used in the center of the output of optical systems that have a shifted input plane, and the optical waves propagation of these systems is described by the FrT.

### 3.2. Convolution Operation

The usual convolution of two functions f(x) and h(x) under the Fourier transform (FT) is computed in the Fourier domain (FD) by using the product of the FTs of these functions f(x) and h(x). In order to define a new convolution operation with a similar property under the FrT, we propose the following integral convolution operation for parameters λ and *z*:

(9) $$f\left(x\right){*}_{\lambda ,z}h\left(x\right)=M_{\lambda ,z}\int_{-\infty }^{+\infty }f\left(x^{\prime }\right)h\left(x-x^{\prime }\right)\exp\left\{\frac{-i2\pi x^{\prime }}{\lambda z}\left(x-x^{\prime }\right)\right\}dx^{\prime }.$$

The computation of the new convolution operation in the FrD for the parameters λ and *z* is:

(10) $${\mathrm{FrT}}_{\lambda ,z}\left\{f\left(x\right){*}_{\lambda ,z}h\left(x\right)\right\}=f_{z}\left(u\right)\left[h_{z}\left(u\right)\exp\left\{\frac{-i\pi u^{2}}{\lambda z}\right\}\right],$$

where $f_{z}\left(u\right)={\mathrm{FrT}}_{\lambda ,z}\left\{f\left(x\right)\right\}$ and $h_{z}\left(u\right)={\mathrm{FrT}}_{\lambda ,z}\left\{h\left(x\right)\right\}$. Therefore, the new convolution operation can be computed using the product of the FrTs of the functions f(x) and h(x) and a quadratic phase term. If the parameter *z* is too large or tends to infinity, the FrD in the Fraunhofer domain and Equation (9) is reduced to the usual convolution, $f\left(x\right){*}_{\lambda ,z}h\left(x\right)=f\left(x\right)*h\left(x\right)$. The result of the new convolution operation between a function f(x) and a shifted Dirac delta function $\delta (x-x_{0})$ is given by:

(11) $$f\left(x\right){*}_{\lambda ,z}\delta \left(x-x_{0}\right)=M_{\lambda ,z}\exp\left\{\frac{-i2\pi x_{0}}{\lambda z}\left(x-x_{0}\right)\right\}f\left(x-x_{0}\right),$$

the result of the previous equation allows us to express the new generalized shift operation presented in Section 3.1 as follows:

(12) $${\mathrm{GS}}_{x_{0};\lambda ,z}f\left(x\right)=\frac{1}{M_{\lambda ,z}}\left[f\left(x\right){*}_{\lambda ,z}\delta \left(x-x_{0}\right)\right]\exp\left\{\frac{-i\pi x_{0}^{2}}{\lambda z}\right\}.$$

The new convolution operation based on the FrT is invariant to the new generalized shift operation:

(13) $${\mathrm{GS}}_{x_{0};\lambda ,z}\left[f\left(x\right){*}_{\lambda ,z}h\left(x\right)\right]=\left[{\mathrm{GS}}_{x_{0};\lambda ,z}f\left(x\right)\right]{*}_{\lambda ,z}h\left(x\right)=f\left(x\right){*}_{\lambda ,z}\left[{\mathrm{GS}}_{x_{0};\lambda ,z}h\left(x\right)\right].$$

### 3.3. Correlation Operation

Our motivation to define a new correlation operation is very similar to the purpose described in the first paragraph of the previous subsection. Therefore, we define a new correlation operation for parameters λ and *z* by using the following integral expression:

(14) $$f\left(x\right){⊛}_{\lambda ,z}h\left(x\right)=M_{\lambda ,z}^{*}\int_{-\infty }^{+\infty }f\left(x^{\prime }\right)h^{*}\left(x^{\prime }-x\right)\exp\left\{\frac{i2\pi x}{\lambda z}\left(x^{\prime }-x\right)\right\}dx^{\prime },$$

where the superscript ${}^{*}$ denotes the complex conjugation operation. This new correlation operation in the FrD for the parameters λ and *z* is represented by:

(15) $${\mathrm{FrT}}_{\lambda ,z}\left\{f\left(x\right){⊛}_{\lambda ,z}h\left(x\right)\right\}=f_{z}\left(u\right){\left[h_{z}\left(u\right)\exp\left\{\frac{-i\pi u^{2}}{\lambda z}\right\}\right]}^{*},$$

where $f_{z}\left(u\right)={\mathrm{FrT}}_{\lambda ,z}\left\{f\left(x\right)\right\}$ and $h_{z}\left(u\right)={\mathrm{FrT}}_{\lambda ,z}\left\{h\left(x\right)\right\}$. The new correlation operation is computed using the product of the FrTs $f_{z}\left(u\right)$ and the complex conjugate of $h_{z}\left(u\right)$ and a quadratic phase term. If the parameter *z* is too large or tends to infinity, the new correlation operation given by Equation (14) is reduced to the usual correlation, $f\left(x\right){⊛}_{\lambda ,z}h\left(x\right)=f\left(x\right)⊛h\left(x\right)$. The new correlation operation based on the FrT is also invariant to the new generalized shift operation:

(16) $${\mathrm{GS}}_{x_{0};\lambda ,z}\left[f\left(x\right){⊛}_{\lambda ,z}h\left(x\right)\right]=\left[{\mathrm{GS}}_{x_{0};\lambda ,z}f\left(x\right)\right]{⊛}_{\lambda ,z}h\left(x\right)=f\left(x\right){⊛}_{\lambda ,z}\left[{\mathrm{GS}}_{-x_{0};\lambda ,z}h\left(x\right)\right].$$

The results of the new definitions for convolution and correlation based on the FrT depend on the information contained in the FrTs of the functions f(x) and h(x), along with a modulation by a pure quadratic phase term, such as that which is shown in Equations (10) and (15). The differences between the results of the new convolution and correlation operations based on the FrT and the usual convolution and correlation operations based on the FT are related to the differences between the near-field optics described by the FrT and the far-field optics described by the FT [1].

## 4. Applications of the New Processing Operations Based on the FrT

### 4.1. Sampling Theorem Based on the FrT

We obtain the sampling theorem by using the FrT and the new processing operations described in the previous section. The sampling theorem is very important for the modern optical implementation of information processing systems that are composed of cameras, spatial light modulators and other optical and optoelectronic devices. The correct sampling at the spatial domain and in the FrD is a critical aspect for the information processing systems based on the free-space optical propagation because the characteristics of optical waves propagation can be preserved or modified when these samplings are defined correctly or incorrectly, respectively.

In order to simplify the mathematical process of obtaining the sampling theorem based on the FrT, we introduce a new Dirac comb function for parameters λ, *z* and period *T*, which is given by:

(17) $$comb_{\lambda ,z}\left(\frac{x}{T}\right)=T\sum_{n=-\infty }^{\infty }\exp\left\{\frac{-i\pi {\left(nT\right)}^{2}}{\lambda z}\right\}\delta \left(x-nT\right).$$

The FrT for the parameters λ and *z* in the previous equation is:

(18) $${\mathrm{FrT}}_{\lambda ,z}\left\{comb_{\lambda ,z}\left(\frac{x}{T}\right)\right\}=M_{\lambda ,z}Tcomb_{\lambda ,-z}\left(\frac{u}{\lambda z/T}\right).$$

The previous equation shows that the FrT for parameters λ and *z* of the proposed Dirac comb function for parameters λ, *z* and period *T* corresponds to the other Dirac comb function for parameters λ, −z and period λz/T. We describe the sampling theorem based on the FrT for a function f(x) whose FrT $f_{z}\left(u\right)$ for parameters λ and *z* has finite support $\left[-u_{0}/2,u_{0}/2\right]$ for coordinate *u* at the FrD. The following expression S(u) denotes the shifted replicas of $f_{z}\left(u\right)$:

(19) $$\begin{array}{cc} S(u)= & f_{z}\left(u\right){*}_{\lambda ,-z}comb_{\lambda ,-z}\left(\frac{u}{u_{0}}\right)\\ = & M_{\lambda ,-z}u_{0}\sum_{n=-\infty }^{\infty }f_{z}\left(u-nu_{0}\right)\exp\left\{\frac{i2\pi nu_{0}u}{\lambda z}\right\}\exp\left\{\frac{-i\pi {\left(nu_{0}\right)}^{2}}{\lambda z}\right\}. \end{array}$$

The FrT for parameters λ and −z of Equation (19) is:

(20) $$S\left(x\right)={\mathrm{FrT}}_{\lambda ,-z}\left\{S(u)\right\}=\frac{1}{M_{\lambda ,z}}\sum_{n=-\infty }^{\infty }f\left(n\frac{\lambda z}{u_{0}}\right)\delta \left(x-n\frac{\lambda z}{u_{0}}\right).$$

The function S(x) of the previous equation represents a sampled version of f(x) with a sampling period that is defined by $\lambda z/u_{0}$. The new convolution operation of Section 3.2 for parameters λ and *z* is utilized to perform the filtering of the function S(x) using a low-pass filter in the FrD. This filtering allows us to reconstruct f(x) from S(x), as follows:

(21) $$\begin{array}{cc} f(x)= & S\left(x\right){*}_{\lambda ,z}{\mathrm{FrT}}_{\lambda ,-z}\left\{\frac{1}{u_{0}}rect\left(\frac{u}{u_{0}}\right)\exp\left\{\frac{i\pi u^{2}}{\lambda z}\right\}\right\}\\ = & M_{\lambda ,-z}\exp\left\{\frac{-i\pi x^{2}}{\lambda z}\right\}\sum_{n=-\infty }^{\infty }\exp\left\{\frac{i\pi {\left(nT_{x}\right)}^{2}}{\lambda z}\right\}f\left(nT_{x}\right)\frac{\sin\left(\pi \left(x-nT_{x}\right)/T_{x}\right)}{\pi \left(x-nT_{x}\right)/T_{x}}, \end{array}$$

where $T_{x}\leq \lambda z/u_{0}$ in order to avoid aliasing for the reconstruction of f(x), and the low-pass filter in the FrD is performed with a rectangle function whose support over the coordinate *u* is $u_{0}$, which corresponds to the finite support of the FrT $f_{z}\left(u\right)$. This last equation denotes the sampling theorem based on the FrT, and this reconstruction equation of f(x) allows us to obtain the values of f(x) for every *x* from the sampled version of f(x) when the sampling period is $T_{x}\leq \lambda z/u_{0}$.

### 4.2. The Double Random Phase Encoding (DRPE) in the FrD

Double random phase encoding (DRPE) is a very important technique used to encrypt images with a high level of security by using an optical system [6,7,8]. DRPE uses two random phase masks (RPMs) to convert (encrypt) an original image into a noisy image (encrypted image). Initially, the DRPE was implemented in the FD using a holographic 4*f*-processor [1]. Later, the optical DRPE was extended from the FD to the FrD [12], the fractional Fourier domain [13] and the Collins domain [14] in order to improve the security of the encryption system. The DRPE can also be optically implemented with a nonlinear joint transform correlator (JTC) architecture in different optical processing domains (such as FD and FrD) with the purpose of increasing the quality of the decrypted image and obtaining better security over the encrypted image and practical setups with no hard alignment requirements [3,4,5,15,16,17,18,19,20].

In this section, we propose a modified version of the DRPE using the new processing operations based on FrT. This modified version of the DRPE in the FrD introduces new security keys that are given by the parameters of shift and distance of the proposed generalized shift operation in order to increase the security of the encrypted image. The original image to encrypt is a real-valued grayscale image f(x) with values in the interval [0, 1]. We use two RPMs for the encryption-decryption system, which are given by:

(22) $$r\left(x\right)=\exp\left\{i2\pi m\left(x\right)\right\},\;h_{z_{1}}\left(u\right)=\exp\left\{i2\pi n\left(u\right)\right\},$$

where *x* and *u* are the coordinates in the spatial domain and the FrD, respectively, and m(x) and n(u) are normalized positive functions that are randomly generated, statistically independent and uniformly distributed in the interval [0, 1] [6,15,16].

We define the encrypted image by using the original image f(x) that is encoded in amplitude, the two RPMs r(x) and $h_{z_{1}}\left(u\right)$, and the proposed generalized shift and convolution operations. The first step of the encryption system is:

(23) $$\begin{array}{cc} s_{z_{1}}\left(u\right)= & {\mathrm{FrT}}_{\lambda ,z_{1}}\left\{{\mathrm{GS}}_{x_{1};\lambda ,z_{1}}\left[f(x)r(x)\right]{*}_{\lambda ,z_{1}}{\mathrm{GS}}_{x_{2};\lambda ,z_{1}}\left[h(x)\right]\right\}\\ = & \exp\left\{\frac{-i2\pi x_{1}u}{\lambda z_{1}}\right\}p_{z_{1}}\left(u\right)\left[\exp\left\{\frac{-i2\pi x_{2}u}{\lambda z_{1}}\right\}h_{z_{1}}\left(u\right)\exp\left\{\frac{-i\pi u^{2}}{\lambda z_{1}}\right\}\right], \end{array}$$

where $p_{z_{1}}\left(u\right)={\mathrm{FrT}}_{\lambda ,z_{1}}\left\{f\left(x\right)r\left(x\right)\right\}$ and $h_{z_{1}}\left(u\right)={\mathrm{FrT}}_{\lambda ,z_{1}}\left\{h\left(x\right)\right\}$. In this first step of the encryption process, the original image to needs to be encrypted f(x) is encoded in the amplitude and multiplied by the RPM r(x) on the input plane of the encryption system. Then, this product of f(x)r(x) is shifted to $x_{1}$ and modulated by a pure linear phase term by means of the generalized shift operation ${\mathrm{GS}}_{x_{1};\lambda ,z_{1}}$. We perform the convolution for parameters λ and $z_{1}$ in the first FrD for the parameters λ and $z_{1}$ between the shifted and modulated product f(x)r(x) and the distribution h(x), which is shifted to $x_{2}$ and modulated by another pure linear phase. This convolution in the first FrD for the parameters λ and $z_{1}$ is computed by the product between the Fresnel propagation (FrT) of the distribution ${\mathrm{GS}}_{x_{1};\lambda ,z_{1}}\left[f\left(x\right)r\left(x\right)\right]$ at distance $z_{1}$ when this distribution is illuminated with a plane wave of wavelength λ, and the distribution given by the multiplication of the RPM $h_{z_{1}}\left(u\right)$, a pure linear phase term and a quadratic phase term. The data distribution ${\mathrm{GS}}_{x_{1};\lambda ,z_{1}}\left[f\left(x\right)r\left(x\right)\right]$ is placed on the input plane of the encryption system, and its FrT plane, which is the first FrD for the parameters λ and $z_{1}$, is located at distance $z_{1}$ with respect to the input plane of the system.

Equation (23) allows us to place two data distributions centred at different locations on the input plane of the encryption system by using the generalized shift operation; this feature is not possible for the proper working of the DRPE in the FrD presented in [12]. The application of this generalized shift operations on the input plane of the encryption system allow us to ensure the complete overlapping of the data distributions $p_{z_{1}}\left(u\right)$ and $h_{z_{1}}\left(u\right)$ in the FrD for the parameters λ and $z_{1}$. This feature is very important to implement the DRPE technique because the encrypted image will be a noisy image when this feature is fulfilled [3,6,7,8,12,15,16,17,18]. Therefore, the correct implementation of the DRPE in the FrD is achieved due to proposed generalized shift and convolution operations based on the FrT. The proposed new processing operations based on the FrT can be used to describe and analyse the nonlinear encryption systems based on a JTC in the FrD in a compact form [3,19,20].

The second step of the encryption system is to perform the FrT for parameters λ and $z_{2}$ over the data distribution $s_{z_{1}}\left(u\right)$:

(24) $$E_{z_{2}}\left(x^{\prime }\right)={\mathrm{FrT}}_{\lambda ,z_{2}}\left\{s_{z_{1}}\left(u\right)\right\}.$$

The previous equation represents a Fresnel propagation (FrT) of the distribution $s_{z_{1}}\left(u\right)$ from the first FrD for parameters λ and $z_{1}$ to the distance $z_{2}$ with the same wavelength λ. The resulting data distribution of Equation (24) is located in the second FrD for parameters λ and $z_{2}$. Finally, the encrypted image is defined by:

(25) $${\tilde{E}}_{z_{2}}\left(x^{\prime }\right)={\mathrm{GS}}_{x_{3};\lambda ,z_{3}}\left\{E_{z_{2}}\left(x^{\prime }\right)\right\}=\exp\left\{\frac{-i2\pi x_{3}}{\lambda z_{3}}\left(x^{\prime }-\frac{x_{3}}{2}\right)\right\}E_{z_{2}}\left(x^{\prime }-x_{3}\right).$$

**Figure 1:**
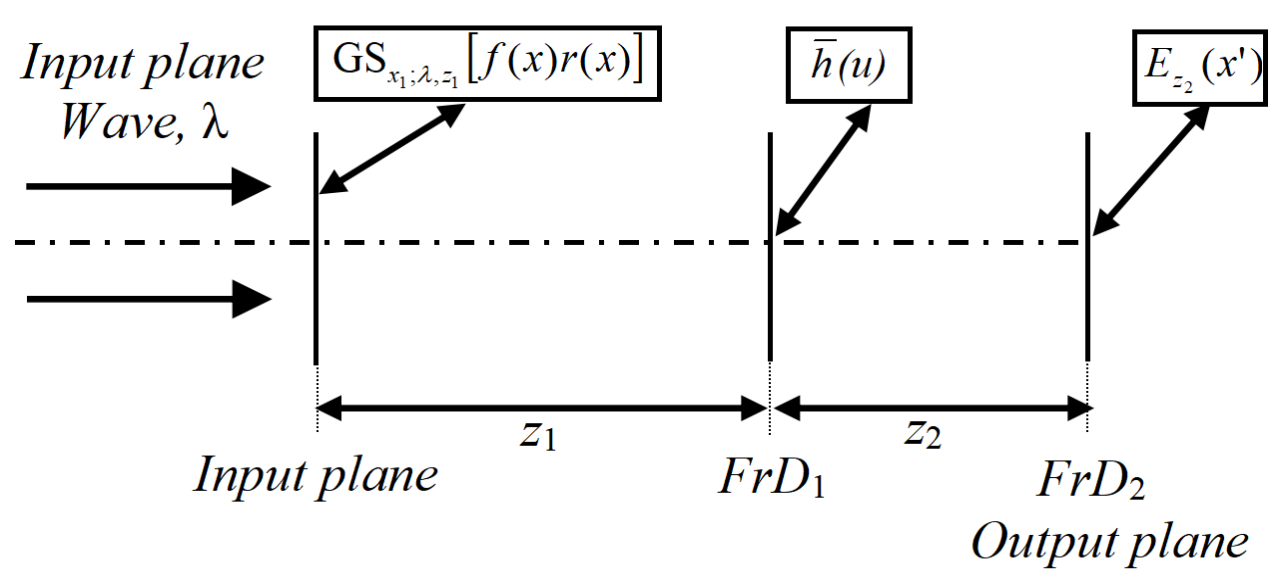
Optical schematic diagram of the proposed encryption system, where the data distribution in the first FrD is $\bar{h}\left(u\right)=\exp\left\{-i2\pi x_{2}u/\lambda z_{1}\right\}h_{z_{1}}\left(u\right)\exp\left\{-i\pi u^{2}/\lambda z_{1}\right\}$.

The encrypted image ${\tilde{E}}_{z_{2}}\left(x^{\prime }\right)$ is defined in the second FrD for parameters λ and $z_{2}$, and it corresponds to image $E_{z_{2}}\left(x^{\prime }\right)$ that is shifted to $x_{3}$ and modulated by a pure linear phase term. The six security keys of the encryption system are given by the RPM $h_{z_{1}}\left(u\right)$, the wavelength λ and the distances $z_{1}$, $z_{2}$, $z_{3}$ and $x_{3}$. The security keys for the encryption system of this subsection, apart from the RPM key, are added because we used the new processing operations based on the FrT. The proposed encryption system introduces two new security keys given by the distances $z_{3}$ and $x_{3}$ with respect to the previous DRPE in the FrD [12]. When the shifts $x_{1}$, $x_{2}$ and $x_{3}$ are equal to zero, the proposed encryption of this paper is very similar to the DRPE in the FrD presented in [12]. Figure 1 shows the optical encryption scheme based on the FrT and the new processing operations proposed in this work.

The correct output of the decryption system, which is the correct decrypted image, is obtained from the encrypted image ${\tilde{E}}_{z_{2}}\left(x^{\prime }\right)$ and the six security key RPMs ($h_{z_{1}}\left(u\right)$, λ, $z_{1}$, $z_{2}$, $z_{3}$ and $x_{3}$). The first step of the decryption system is to apply the generalized shift operation with parameters ${\hat{x}}_{3}$, $\hat{\lambda }$ and ${\hat{z}}_{3}$ to the encrypted image ${\tilde{E}}_{z_{2}}\left(x^{\prime }\right)$

(26) $$\begin{array}{cc} {\hat{E}}_{z_{2}}\left(x^{\prime }\right)= & {\mathrm{GS}}_{{\hat{x}}_{3};\hat{\lambda },{\hat{z}}_{3}}\left\{{\tilde{E}}_{z_{2}}\left(x^{\prime }\right)\right\}=\exp\left\{\frac{-i2\pi {\hat{x}}_{3}}{\hat{\lambda }{\hat{z}}_{3}}\left(x^{\prime }-\frac{{\hat{x}}_{3}}{2}\right)\right\}{\tilde{E}}_{z_{2}}\left(x^{\prime }-{\hat{x}}_{3}\right)\\ = & \exp\left\{\frac{-i2\pi {\hat{x}}_{3}}{\hat{\lambda }{\hat{z}}_{3}}\left(x^{\prime }-\frac{{\hat{x}}_{3}}{2}\right)\right\}\exp\left\{\frac{-i2\pi x_{3}}{\lambda z_{3}}\left(x^{\prime }-{\hat{x}}_{3}-\frac{x_{3}}{2}\right)\right\}E_{z_{2}}\left(x^{\prime }-x_{3}-{\hat{x}}_{3}\right)\\ = & \exp\left\{\frac{i2\pi }{\hat{\lambda }{\hat{z}}_{3}}\left(1-a\right)ax_{3}^{2}\right\}\exp\left\{\frac{-i2\pi x_{5}}{\hat{\lambda }{\hat{z}}_{3}}\left(x^{\prime }-\frac{x_{5}}{2}\right)\right\}E_{z_{2}}\left(x^{\prime }-x_{6}\right), \end{array}$$

where $a=\left(\hat{\lambda }{\hat{z}}_{3}\right)/\left(\lambda z_{3}\right)$, $x_{5}={\hat{x}}_{3}+ax_{3}$ and $x_{6}={\hat{x}}_{3}+x_{3}$. In order to retrieve the data distribution $E_{z_{2}}\left(x^{\prime }\right)$ of Equation (24) without performing shift and modulation by using a pure linear phase term from ${\hat{E}}_{z_{2}}\left(x^{\prime }\right)$, the correct three security keys used in the first step of decryption system have to be set exactly as ${\hat{x}}_{3}=-x_{3}$, $\hat{\lambda }=\lambda$ and ${\hat{z}}_{3}=z_{3}$. The second step of the decryption system is to apply the FrT for parameters $\hat{\lambda }$ and $-{\hat{z}}_{2}$ to the data distribution ${\hat{E}}_{z_{2}}\left(x^{\prime }\right)$

(27) $${\hat{s}}_{{\hat{z}}_{1}}\left(u\right)={\mathrm{FrT}}_{\hat{\lambda },-{\hat{z}}_{2}}\left\{{\hat{E}}_{z_{2}}\left(x^{\prime }\right)\right\}.$$

In this second step of the decryption system, we use two security keys that are given by $\hat{\lambda }$ and ${\hat{z}}_{2}$. If the following security keys are set by ${\hat{x}}_{3}\neq -x_{3}$, $\hat{\lambda }\neq \lambda$, ${\hat{z}}_{3}\neq z_{3}$ and ${\hat{z}}_{2}\neq z_{2}$, the result of Equation (27) that is given by ${\hat{s}}_{{\hat{z}}_{1}}\left(u\right)$ would be very different to the $s_{z_{1}}\left(u\right)$ of Equation (23), and thus, the decryption process will be unsuccessful because the decrypted image will have a noisy distribution. For the case when ${\hat{x}}_{3}\neq -x_{3}$, $\hat{\lambda }=\lambda$, ${\hat{z}}_{3}\neq z_{3}$ and ${\hat{z}}_{2}=z_{2}$, the result of Equation (27) is:

(28) $$\begin{array}{cc} {\hat{s}}_{{\hat{z}}_{1}}\left(u\right)= & {\mathrm{FrT}}_{\lambda ,-z_{2}}\left\{{\hat{E}}_{z_{2}}\left(x^{\prime }\right)\right\}\\ = & K\exp\left\{\frac{-i2\pi }{\lambda {\hat{z}}_{3}}\left({\hat{x}}_{3}+\frac{{\hat{z}}_{3}}{z_{3}}x_{3}\right)u\right\}s_{z_{1}}\left(u-\left(1+\frac{z_{2}}{{\hat{z}}_{3}}\right){\hat{x}}_{3}-\left(1+\frac{z_{2}}{z_{3}}\right)x_{3}\right), \end{array}$$

where *K* is a complex-valued constant. The result of Equation (28) is a shifted and modulated version of the data distribution $s_{z_{1}}\left(u\right)$, which is defined in Equation (23). Now, if ${\hat{x}}_{3}=-x_{3}$, $\hat{\lambda }=\lambda$, ${\hat{z}}_{3}=z_{3}$ and ${\hat{z}}_{2}=z_{2}$, Equation (28) is reduced to ${\hat{s}}_{{\hat{z}}_{1}}\left(u\right)=s_{z_{1}}\left(u\right)$. The third step of the decryption system is to compute the following product:

(29) $$\begin{array}{cc} t_{{\hat{z}}_{1}}\left(u\right)= & {\hat{s}}_{{\hat{z}}_{1}}\left(u\right){\left[\exp\left\{\frac{-i2\pi x_{2}u}{\lambda {\hat{z}}_{1}}\right\}{\hat{h}}_{{\hat{z}}_{1}}\left(u\right)\exp\left\{\frac{-i\pi u^{2}}{\lambda {\hat{z}}_{1}}\right\}\right]}^{*}\\ = & {\mathrm{FrT}}_{\lambda ,{\hat{z}}_{1}}\left\{\hat{s}\left(x\right){⊛}_{\lambda ,{\hat{z}}_{1}}{\mathrm{GS}}_{x_{2};\lambda ,{\hat{z}}_{1}}\left[\hat{h}\left(x\right)\right]\right\}, \end{array}$$

where ${\mathrm{FrT}}_{\lambda ,{\hat{z}}_{1}}\left\{\hat{s}\left(x\right)\right\}={\hat{s}}_{{\hat{z}}_{1}}\left(u\right)$ and ${\mathrm{FrT}}_{\lambda ,{\hat{z}}_{1}}\left\{\hat{h}\left(x\right)\right\}={\hat{h}}_{{\hat{z}}_{1}}\left(u\right)\neq h_{z_{1}}\left(u\right)$. Equation (29) is the proposed correlation in the FrD for the parameters λ and ${\hat{z}}_{1}$ between the data distributions of $\hat{s}\left(x\right)$ and ${\mathrm{GS}}_{x_{2};\lambda ,{\hat{z}}_{1}}\left[\hat{h}\left(x\right)\right]$. In this third step of the decryption system, we use the security keys corresponding to ${\hat{z}}_{1}$ and ${\hat{h}}_{{\hat{z}}_{1}}\left(u\right)$. If the following keys are set by ${\hat{x}}_{3}\neq -x_{3}$, $\hat{\lambda }=\lambda$, ${\hat{z}}_{3}\neq z_{3}$, ${\hat{z}}_{2}=z_{2}$, ${\hat{z}}_{1}=z_{1}$ and ${\hat{h}}_{{\hat{z}}_{1}}\left(u\right)=h_{z_{1}}\left(u\right)$, Equation (29) will never be reduced to the data distribution $\left\{-i2\pi x_{1}u/\lambda z_{1}\right\}p_{z_{1}}\left(u\right)$. Finally, when the six security keys are correct in the decryption system, i.e., ${\hat{x}}_{3}=-x_{3}$, $\hat{\lambda }=\lambda$, ${\hat{z}}_{3}=z_{3}$, ${\hat{z}}_{2}=z_{2}$, ${\hat{z}}_{1}=z_{1}$ and ${\hat{h}}_{{\hat{z}}_{1}}\left(u\right)=h_{z_{1}}\left(u\right)$, the decrypted image is obtained as follows:

(30) $$\begin{array}{cc} \hat{f}\left(x-x_{1}\right)= & \left|{\mathrm{FrT}}_{\lambda ,-z_{1}}\left\{t_{{\hat{z}}_{1}}\left(u\right)\right\}\right|=\left|{\mathrm{FrT}}_{\lambda ,-z_{1}}\left\{\exp\left\{\frac{-i2\pi x_{1}u}{\lambda z_{1}}\right\}p_{z_{1}}\left(u\right)\right\}\right|\\ = & \left|{\mathrm{GS}}_{x_{1};\lambda ,z_{1}}\left[f(x)r(x)\right]\right|=f\left(x-x_{1}\right). \end{array}$$

The decrypted image $\hat{f}\left(x\right)$ will be a replica of the original image f(x) when the six security keys in the decryption system are correct. If one or more of the six keys are wrong, the decrypted image will have a noisy data distribution. Therefore, the correct retrieval of the original image at the output of the decryption system is only possible when all of the values of the six security keys used in the decryption system are the same values values that were used in the encryption system. The optical schematic diagram of Figure 1 can be used to implement the proposed decryption system by using the complex conjugate of $E_{z_{2}}\left(x^{\prime }\right)$ on the input plane of this optical schematic [12]. The design, description and analysis of the proposed encryption and decryption systems were performed by using the new processing operations proposed in this work. The use of these new processing operations produces a simple and compact characterization and implementation of the behaviour of the proposed modified version of the DRPE in the FrD of this subsection.

**Figure 2:**
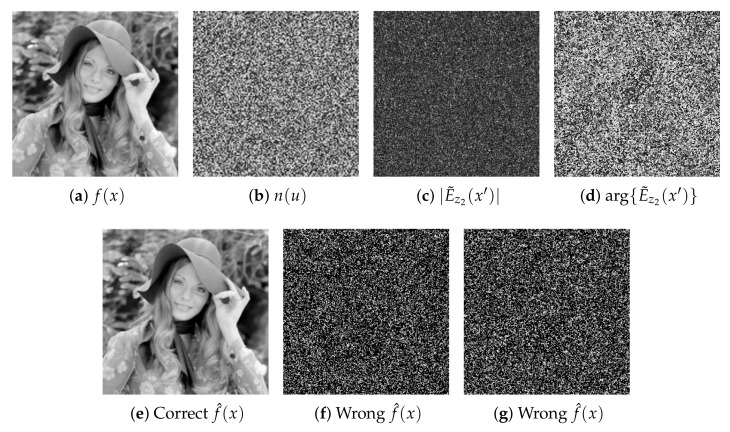
(**a**) Original image to encrypt f(x). (**b**) Random code image n(u) of the RPM $h_{z_{1}}\left(u\right)$. (**c**) Amplitude and (**d**) phase of the encrypted image ${\tilde{E}}_{z_{2}}\left(x^{\prime }\right)$ using the following values of the security keys λ=532 nm, $z_{1}=70$ mm, $z_{2}=82$ mm, $z_{3}=22$ mm and $x_{3}=10$ mm. (**e**) Decrypted image $\hat{f}\left(x\right)$ using the correct values of the six security keys ($h_{z_{1}}\left(u\right)$, λ, $z_{1}$, $z_{2}$, $z_{3}$ and $x_{3}$). Decrypted images for the following wrong security keys: (**f**) the ${\hat{z}}_{3}=21.877\;m\neq z_{3}$, and (**g**) the RPM ${\hat{h}}_{{\hat{z}}_{1}}\left(u\right)\neq h_{z_{1}}\left(u\right)$, when the other five security keys are correct.

Figure 2 shows the computational results for the proposed encryption and decryption systems based on the generalized shift, convolution and correlation operations in the FrD. The images in Figure 2a,b represent the original image that needs to be encrypted f(x) and the random code image n(u) of the RPM $h_{z_{1}}\left(u\right)$, respectively. The random code image m(x) of the RPM r(x) is very similar in appearance to the image depicted in Figure 2b, but the values of random image m(x) are different from the values of random image n(u). The amplitude and phase of the encrypted image ${\tilde{E}}_{z_{2}}\left(x^{\prime }\right)$ for the keys λ=532 nm, $z_{1}=70$ mm, $z_{2}=82$ mm, $z_{3}=22$ mm and $x_{3}=10$ mm, are shown in Figure 2c and Figure 2d, respectively. The right decrypted image $\hat{f}\left(x\right)$ is presented in Figure 2e, where the correct values of the six security keys are provided to the decryption system. If the wrong value of the security key, such as ${\hat{z}}_{3}=21.877\;m\neq z_{3}$, or ${\hat{h}}_{{\hat{z}}_{1}}\left(u\right)\neq h_{z_{1}}\left(u\right)$, is introduced in the decryption system with the other correct five security keys, the resulting decrypted images will be the noisy images that are depicted in Figure 2f and Figure 2g, respectively.

### 4.3. Possible Case Studies Based on the New Processing Operators of This Work

In our research group, we numerically simulate new phase elements for photovoltaic cells and solar collectors with the purpose of focusing the thermal energy irradiated from the sun. This focusing of the sun’s rays can be performed with new phase elements based on the generalized shift operation proposed in this work. We are designing new phase elements that allow the sun’s rays to be focused on the same point or region, regardless of their orientation when they enter Earth’s atmosphere in order to eliminate the heavy mechatronic solar tracking systems.

The FrT is very important for optical processing systems because this optical transform allows us to implement centred, compact and lens-less optical systems for near-field optics [3,19,20]. Therefore, the new processing operators in this work can describe and analyse these optical processing system in the near-field regime. For instance, the JTC is an optical architecture that can be used for holography, target recognition, object distance detection, video surveillance, authentication, encryption, decryption and DNA sequence alignment, among other applications. The JTC in the FrD allows us to implement compact and lens-less optical systems when the generalized shift operation of this work is applied to each data distribution of the input plane of the JTC because all of the resulting data distributions on the output plane of the JTC in the FrD, which are contained in the joint Fresnel power distribution (JFPD) [3], are centred at the same place. The different terms of the JFPD can be easily described and analysed by the proposed convolution and correlation operations in this work. This description and analysis of the JTC in the FrD will be simple and compact, which would allow researchers to suitably study of the behaviour for the optical processing systems based on the JTC architecture in the FrD.

Finally, we will expect that several optical processing systems in the FrD for applications in holography, imaging systems, microscopy, diffractive optical elements, spectroscopy, beam focusing, surface modification, optical tweezers, security systems and image filtering systems, among others applications, will be designed, implemented, analysed and improved by using the new processing operators proposed in this work.

## 5. Conclusions

New processing operations based on the FrT have been proposed. The new processing operations are the generalized shift, the convolution and the correlation of the FrD. The generalized shift operation simultaneously produces a shift and modulation in the pure linear phase of a given function, and the FrT of this generalized shift operation allows us to obtain a function in the FrD without the shift. This fact of the generalized shift operation is very important for centred optical systems in the FrD, such as holographic ones, optical image formation and optical security systems, among others. The new integral expressions for the proposed convolution and correlation operations are generalized versions of the usual convolution and correlation operations, and these operations can be computed at the spatial domain for different values of the parameters λ and *z*. The proposed convolution and correlation operations of this work are performed in the FrD by means of the product of two FrTs and a quadratic phase term. We have developed two applications for the new processing operations based on the FrT. The first application was the sampling theorem based on the FrT for a function, whose resulting FrT has a finite support. We defined a new Dirac comb function for parameters λ, *z* and *T*, and we also used the new convolution operation in this paper in order to obtain a reconstruction formula for the sampling theorem based on the FrT. We have proved that the sampling period at the spatial domain of a function is directly proportional to the product of λz and inversely proportional to the length of the finite support for the resulting FrT of the function that needs to be sampled. The second application was to propose and describe a modified version of the DRPE in the FrD by using all of the proposed new processing operations. The proposed encryption system in the FrD introduced two new security keys related to the parameters of the generalized shift operation, which allows us to improve the security of the encrypted image. The new processing operations proposed in this paper can also be used to describe and analyse other optical architectures of the DRPE technique. Finally, the new processing operations in this work could be used to develop new optical information processing systems based on the FrT.

## Data Availability

The supporting information can be found from the corresponding author.

## Abbreviations

The following abbreviations are used in this manuscript:

DRPE	Double random phase encoding
FD	Fourier domain
FrD	Fresnel domain
FT	Fourier transform
FrT	Fresnel transform
JFPD	Joint Fresnel power distribution
JTC	Joint transform correlator
RPMs	Random phase masks
